# Developing a Five-Minute Normative Database of Heart Rate Variability for Diagnosing Cardiac Autonomic Dysregulation for Patients with Major Depressive Disorder

**DOI:** 10.3390/s24124003

**Published:** 2024-06-20

**Authors:** Li-Hsin Chang, Min-Han Huang, I-Mei Lin

**Affiliations:** 1 Department of Psychology, College of Humanities and Social Sciences, Kaohsiung Medical University, Kaohsiung 807378, Taiwan; olivia1999hsin@gmail.com (L.-H.C.); u111565007@gap.kmu.edu.tw (M.-H.H.); 2 Department of Medical Research, Kaohsiung Medical University Hospital, Kaohsiung 807378, Taiwan

**Keywords:** depression, heart rate variability (HRV), Taiwan HRV normative database, Z-scores

## Abstract

Heart rate variability (HRV) is related to cardiac vagal control and emotional regulation and an index for cardiac vagal control and cardiac autonomic activity. This study aimed to develop the Taiwan HRV normative database covering individuals aged 20 to 70 years and to assess its diagnosing validity in patients with major depressive disorder (MDD). A total of 311 healthy participants were in the HRV normative database and divided into five groups in 10-year age groups, and then the means and standard deviations of the HRV indices were calculated. We recruited 272 patients with MDD for cross-validation, compared their HRV indices with the normative database, and then converted them to Z-scores to explore the deviation of HRV in MDD patients from healthy groups. The results found a gradual decline in HRV indices with advancing age in the HC group, and females in the HC group exhibit higher cardiac vagal control and parasympathetic activity than males. Conversely, patients in the MDD group demonstrate lower HRV indices than those in the HC group, with their symptoms of depression and anxiety showing a negative correlation with HRV indices. The Taiwan HRV normative database has good psychometric characteristics of cross-validation.

## 1. Introduction

Heart rate variability (HRV) is a noninvasive measurement through electrocardiographic (ECG) and photoplethysmography (PPG) sensors, calculated from the intervals between successive heartbeats, and commonly used as indices of autonomic nervous system activity and cardiac activity [1]. Elevated HRV is often associated with effective emotional regulation and overall health [2,3,4], while reduced HRV tends to be associated with various physical illnesses and mental disorders, including cardiovascular disease and depression disorders [2,5,6]. ECG sensors capture the raw cardiac signals (PQRST), identifying the R-spike to R-spike intervals, whereas PPG sensors detect the peak-to-peak variations in the pulse waveform. Researchers utilized these data to compute interbeat intervals over a period, commonly five minutes for short-term measurement, which are converted into HRV indices [7]. 

In a comprehensive review, Shaffer et al. [8] indicated HRV metrics and an HRV normative database for 24 h, short-term (five minutes), and ultra-short-term (less than five minutes) from previous studies, and pointed out that normative values are not interchangeable across these time frames. Shaffer et al. [8] emphasized the importance of research and clinical professionals tailoring HRV assessment and intervention according to their target populations’ specific characteristics and norms. 

Several normative databases of HRV across different age groups and cultures were published to monitor autonomic nervous system activity. Table 1 illustrates some studies that collected normative databases from various countries. Nunan et al. [9] analyzed HRV indices from five minutes of short-term measurement spanning 1997 to 2008. They meticulously reviewed 44 articles from 3141 research papers, focusing on participants aged 18 and above, with sample sizes exceeding 30, and ECG or PPG was measured for at least five minutes. Their analysis encompassed 21,438 participants and revealed that males exhibited higher SDNN, RMSSD, LF/HF ratio, and LFn.u., alongside lower LF and HF, than females. Abhishekh et al. [10] conducted a study in India, recruiting 189 healthy participants (114 males and 75 females) aged 16–60. They measured a five-minute ECG with respiratory rates between 12 and 15 breaths per minute. Subsequently, they converted ECG data into HRV indices. Their findings indicated negative correlations between age and SDNN, RMSSD, lnHF, and total power, whereas lnLF exhibited a positive correlation with age. Baek et al. [11] also collected data from 467 healthy participants aged between 8 and 69 years from South Korea. Five-minute resting-state PPG on the right index finger was measured using Freeze-Framer (HeartMath LLC, Boulder Creek, CA, USA). The results revealed SDNN and RMSSD were 44.73 ± 27.51 ms and 26.68 ± 18.95 ms, respectively.

In a recent study, Choi et al. [12] enrolled 291 healthy participants (144 males and 147 females) aged between 19 and 69 years in Korea. Participants underwent five-minute resting-state PPG using a device placed on the left index finger (Pulse Analyzer, KFDA Certification No. 11-1296, LAXTHA Inc., Daejeon, South Korea), with measurements taken between 9:00 and 18:00 under natural breathing rates. PPG signals were then transferred into HRV indices. The findings revealed a consistent decrease in all HRV indices (SDNN, HRV index, pNN50, VLF, LF, HF, and TP) with advancing age among healthy Koreans. Additionally, females exhibited lower lnLF than males in the 40–49 age group, while females demonstrated higher HF than males in the 50–59 age group. Ortega et al. [13] recruited 2143 healthy participants (974 males and 1169 females) aged between 10 and 89 years in Singapore. Participants underwent a five-minute resting-state PPG measurement on the right index finger, which was then converted into HRV indices. The results indicated a gradual decline in the SDNN and RMSSD of HRV indices with advancing age. Specifically, the overall RMSSD was 42.4 ± 17.0 ms, and the overall SDDN was 52.0 ± 19.6 ms. 

HRV is related to cardiac vagal control and emotional regulation in patients with major depressive disorder (MDD). Porges [14] proposed the polyvagal theory to elucidate the complex interplay between cardiac autonomic function, emotional regulation, and social interaction behavior. Previous studies demonstrated that patients with MDD exhibited diminished vagal modulation at rest [15,16,17,18,19] as well as during laboratory-induced emotion tasks, such as viewing sad films. A meta-analysis involving 43 studies encompassing 2359 patients with depression and 3547 healthy controls revealed that patients with depression had lower HRV indices (SDNN, RMSSD, pNN50, LF, and HF) compared to the healthy controls; however, no significant difference was observed in the LF/HF ratio between the two groups [19]. Rottenberg [17,20] and Porges [14] indicated that reduced HRV is associated with decreased flexibility and adaptability of the cardiac vagal control. Dysfunction in the ventral vagal complex may contribute to social engagement difficulties, flattened facial expressions, inflexibility in coning with environmental demands, tendencies toward social withdrawal, diminished gaze behavior, and eye contact, all of which are common features of depression [14,20]. While numerous countries have developed their HRV normative databases, only some studies have explored their reliability and validity. This study aimed to develop the Taiwan HRV normative database covering individuals aged 20 to 70 years and to assess its reliability and validity by utilizing a group of patients with major depressive disorder.

## 2. Materials and Methods

### 2.1. Participants

Participants were recruited from 2013 to 2024 and comprised a healthy control group (HC group) and patients with MDD (MDD group). 

In the HC group, participants were recruited from the Health Management Center of Kaohsiung Medical University Hospital, Kaohsiung Medical University campus, the community of Kaohsiung City, and through online flyers. The inclusion criteria included as follows: (1) participants’ health examination reports showed no severe physical disorder (e.g., cancer, kidney disease, stroke, etc.) or psychiatric disorders (e.g., depressive disorder, anxiety disorder, bipolar disorder, schizophrenia, etc.), and they were not taking prescribed medications; (2) total scores on the Beck Depression Inventory-II (BDI–II) and Beck Anxiety Inventory (BAI) were lower than 14 and 8, respectively; and (3) aged between 20 and 70 years old. 

In the MDD group, participants were recruited for cross-validation by the Department of Psychiatry of three medical centers of Kaohsiung Medical University Hospital. The inclusion criteria included as follows: (1) diagnosed with MDD by psychiatrists based on the Diagnostic and Statistical Manual of Mental Disorders, 5th edition (DSM−5; American Psychiatry Association, 2013); (2) total scores on the BDI-II and BAI exceeding 14 and 8, respectively; (3) aged between 20 and 70 years; and (4) without severe physical illnesses or psychiatric disorders. 

The HC group was divided into five age groups: 20–29, 30–39, 40–49, 50–49, and 60–69 years old, with each group comprising a minimum of 15 males and 15 females to establish a normative database. However, only 11 males were enrolled in the 61–70 age group of the HC cohort due to the challenge of finding participants without physical illnesses or mental disorders in this age range. A total of 356 healthy participants were recruited for the study, with 26 participants excluded (age < 20 years [*n* = 1], age > 70 years [*n* = 4], BDI-II or BAI higher than 14 or 8 [*n* = 14], presence of a physical illness [*n* = 7]). Of the remaining 330 healthy participants who completed the HRV measurement, 19 participants were removed from the statistical analysis (issues during ECG measurement [*n* = 14], arrhythmia, or ECG artifacts [*n* = 5]). Ultimately, 311 healthy participants were included in the HC group, comprising 137 males and 174 females.

In the MDD group, 422 patients were referred by psychiatrists, and 118 were excluded (age < 20 years [*n* = 7], age > 70 years [*n* = 3], BDI-II and BAI scores not exceeding 14 and 8 [*n* = 97], primary diagnosis not being MDD [*n* = 2], comorbid with severe physical illnesses [*n* = 8], or daytime sleepiness [*n* = 1]). A total of 304 participants in the MDD group completed the ECG measurement. Thirty-two participants had their HRV data removed from the statistical analysis (issues during ECG measurement [*n* = 12], missing HRV data or questionnaires [*n* = 14], arrhythmia [*n* = 6]). Ultimately, 272 participants (58 males and 163 females) were included in the MDD group. 

This study received approval from the Institutional Review Board of Kaohsiung Medical University Hospital, Taiwan (KMUH IRB-20120209 II, KMUH IRB-FI20160027, and KMUH IRB-F(I) 20200117), Kaohsiung Chang Gung Memorial Hospital, Taiwan (CGMH IRB1604250002), and Kaohsiung Tsyr Huey Hospital, Taiwan (THMH-RES-21051401). Participants provided informed consent before the study and received TWD 500 (approximately USD 16) for their participation.

### 2.2. Material

Demographic data (age and sex), medications used, and scores of the BDI-II and BAI were collected. The BDI-II comprises a 21-item Likert scale designed to assess depressive symptoms, with a total score ranging from 0 to 63 and further divided into cognitive and somatic depression subscales. BDI-II scores below 13 indicate a normal range of depression, 14–19 indicate mild depression, 20–28 indicated moderate depression, and scores above 29 indicate severe depression [21]. Its Chinese version, translated by Chen [22], demonstrated good psychometric properties, including a Cronbach’s α of 0.94, split-half reliability of 0.91, and a correlation of 0.69 with the Chinese Health Questionnaire of 0.69 [23]. Similarly, the BAI consists of a 21-item Likert scale used to assess anxiety symptoms with a total score ranging from 0 to 63 and divided into cognitive and somatic anxiety subscales. BAI scores below 7 indicate a normal range of anxiety, 8–15 indicate mild anxiety, 16–25 indicate moderate anxiety, and scores above 26 indicate severe anxiety [24]. Its Chinese version, translated by Lin [25], exhibited good psychometric qualities, with a Cronbach’s α of 0.95, a split-half reliability of 0.91, and a correlation of 0.72 with the Hamilton Anxiety Scale of 0.72 [26]. 

ECG recording: An ECG sensor with a sampling rate of 2048 Hz (Thought Technology Ltd., Montreal, QC, Canada) was positioned 1cm below the left and right sides of the clavicle, as well as on the skin surface of the fifth rib of the left chest. Lead II ECG was recorded, and ECG raw signals (PQRST wave) were acquired using ProComp Infiniti™ version 6.1.1 (Thought Technology Ltd., Montreal, QC, Canada).

Participants were instructed to refrain from consuming alcohol, coffee, and tea beverages for three hours before the ECG measurement, and recording sessions were scheduled between 9:00 am and 5:00 pm [12,27]. All participants were instructed to sit and rest in a temperature-controlled room (between 24 and 28 °C). The indoor lighting was set to a fixed brightness using 28 W, 95 lm, 6500 K LED white light (TOA, FH28D-EX), and blackout curtains were installed to prevent variations in indoor brightness due to weather changes. ECG signals of all participants were recorded for five minutes in a resting state with their eyes closed in the same laboratory. 

### 2.3. Data Reduction, ECG Processing, and Statistical Analysis

The ECG data were analyzed using the CardioPro HRV Analysis Module (Thought Technology Ltd., Montreal, QC, Canada). Researchers checked the ECG waveform (R-spike) and removed arrhythmia and movement artifacts. Subsequently, interbeat intervals were converted to time and frequency domains of HRV, including the standard deviation of normal-to-normal intervals (SDNN, indicating total HRV), root mean square of the successive normal-to-normal interval differences (RMSSD, indicating vagal activation), low-frequency power (LF; 0.04–0.15 Hz, representing sympathetic and parasympathetic nervous systems coregulation or baroreceptor gain), high-frequency power (HF; 0.15–0.40 Hz, representing parasympathetic nervous system activity), total power (TP; 0.0033–0.4 Hz, representing total HRV), and LF/HF ratio (representing the sympathetic nervous system activity). Due to skewness and kurtosis of the HRV distributions, the LF, HF, LF/HF ratio, and TP were transformed using natural logarithms into lnLF, lnHF, lnLF/HF ratio, and lnTP [8,28].

Statistical analyses were performed using SPSS Statistics 21.0 (IBM Corporation, Armonk, NY, USA). Firstly, this study calculated the mean and standard deviation (SD) of the HRV indices in five age groups for 316 participants in the HC group, and the total Z-scores was 9480 (316 × 6 HRV indices × 5 discrete aged group). Secondly, the Z-scores and absolute value Z-scores (|Z score|) for the MDD group were calculated using the equation (z = (x − μ)/σ) for each discrete HRV indices, and the total number of Z-scores was 8070 (269 × 6 HRV indices × 5 discrete aged group). The Z-scores and |Z score| of the MDD group served as a benchmark for cross-validation. Thirdly, the Student’s *t*-test and chi-square (χ^2^) were used to examine group differences in demographic characteristics. Fourthly, analysis of variance (ANOVA) and analysis of covariance (ANCOVA) for controlling the covariables were used to examine the group differences in HRV indices. Fifthly, Pearson correlations were analyzed for Z-scores, depression, and anxiety in the MDD group. Finally, the R software version 4.2.0 (Free Software Foundation, Boston, MA, USA) with the ggplot2 package was utilized to visualize the HRV indices using the locally weighted scatterplot smoothing method and scatterplots. 

## 3. Results

### 3.1. Participants’ Demographic Characteristics between the HC and MDD Groups

A total of 311 and 272 participants were in the HC and MDD groups, respectively. There was no significant difference in age between the two groups (*t*_(581)_ = −0.349, *p* > 0.05). However, there was a significant difference in sex distribution between the HC and MDD groups (*χ^2^*_(1)_ = 25.030, *p* < 0.001). Specifically, there were fewer males than females in the MDD group compared to the HC group. Moreover, 39.0% (*n* = 103) of patients used serotonin selective inhibitors (SSRIs), 18.93% (*n* = 50) used serotonin–norepinephrine reuptake inhibitors (SNRIs), 4.16% (*n* = 11) used tricyclic antidepressants (TCAs), and 44.69% (*n* = 118) used other antidepressants (Table 2).

After controlling the sex difference, higher total scores for BDI-II, cognitive depression, somatic depression, total scores of BAI, cognitive anxiety, and somatic anxiety were reported in the MDD group compared to the HC group (Table 2). Lower HRV indices were found in the MDD group compared to the HC group, including SDNN, RMSSD, lnLF, lnHF, and lnTP. However, the two groups had no significant difference in the lnLF/HF ratio (Table 2). 

**Figure 1:**
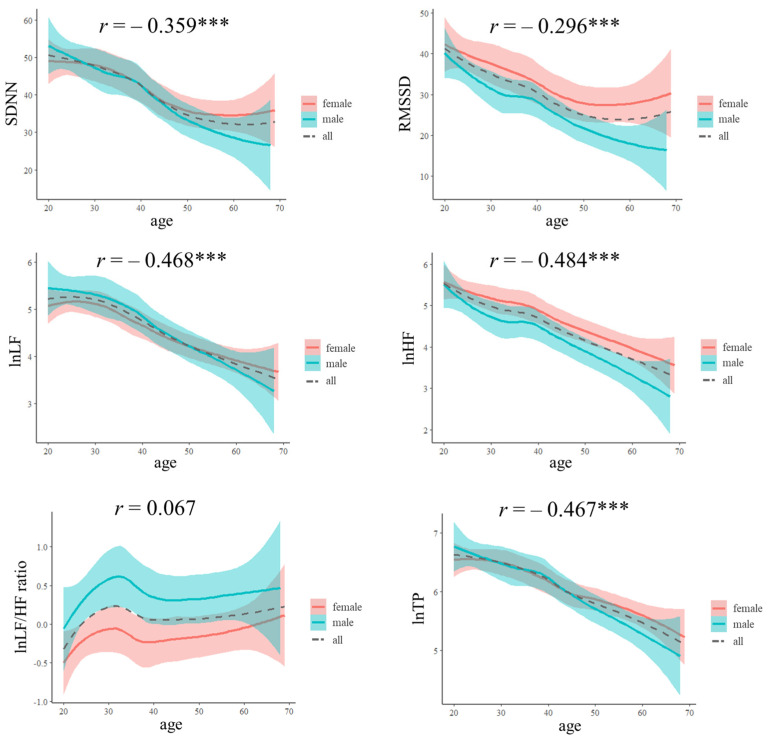
The HRV index across different age groups for male, female, and all participants. Note: The green, red, and gray dashed lines represent the linear regression lines of the HRV index. The shaded green and orange areas depict the 95% confidence interval for HRV in males and females, respectively. *** *p* < 0.001.

**Figure 2:**
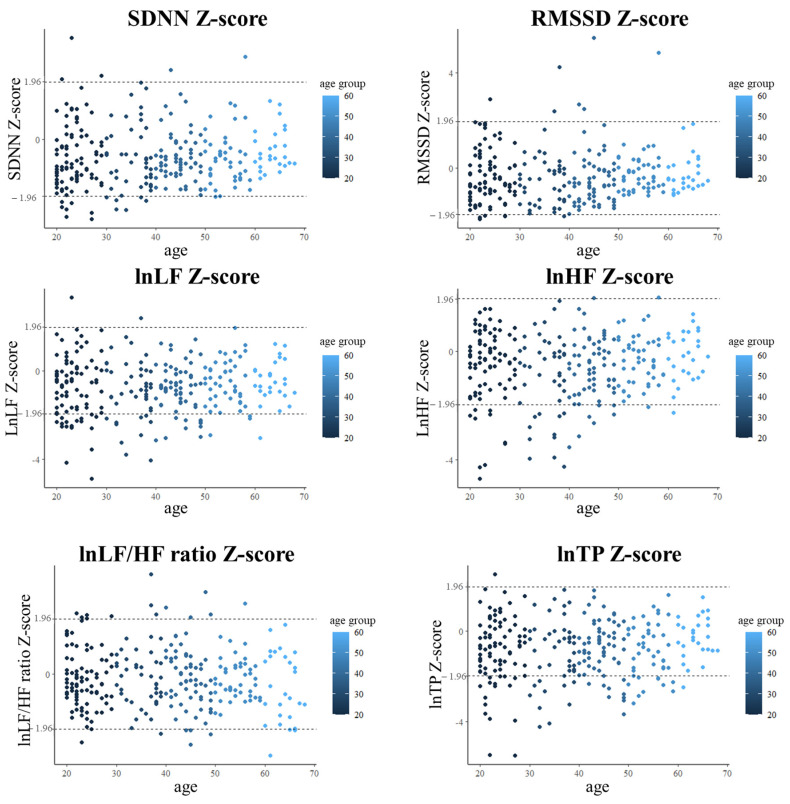
The scatterplots of HRV Z-score in the MDD group.

In the HC group, females exhibited higher RMSSD and lnHF than males (*F*_(1, 309)_ = 10.260, *p* = 0.002; and *F*_(1, 309)_ = 7.657, *p* = 0.006, respectively), and females had a lower lnLF/HF ratio than males (*F*_(1, 309)_ = 19.589, *p* < 0.001). In the MDD group, females exhibited a lower SDNN and lnLF/HF ratio than males (*F*_(1, 270)_ = 5.040, *p* = 0.026; and *F*_(1, 270)_ = 14.865, *p* < 0.001, respectively) (Table 2 and Figure 1). This study also presents scatterplots of the HRV Z-score in the MDD group (Figure 2). As depicted in Figure 2, the Z-scores within the 50–59 and 60–69 age groups fall within the mean ± 1.96 range. Conversely, the Z-scores in younger age groups (20–29, 30–39, 40–49) exceeded this range.

### 3.2. Pearson Correlations between Age, Sex, and HRV Indices in the HC Group

Significant negative correlations were observed between age and SDNN (*r* = −0.359, *p* < 0.0001), RMSSD (*r* = −0.296, *p* < 0.0001), lnLF (*r* = −0.468, *p* < 0.0001), lnHF (*r* = −0.484, *p* < 0.0001), and lnTP (*r* = −0.467, *p* < 0.0001). However, no significant correlations were observed between age and the lnLF/HF ratio (*r* = 0.067, *p* = 0.235) (Figure 1).

### 3.3. Z-Scores and Absolute Value Z-Score across Five Age Groups in the MDD Group

After calculating the Z-score and absolute value Z-score in the MDD group according to the HFV values of the Taiwan HRV normative database, Table 3 presents a Z-score ranging from −0.92 to 1.28 and an absolute value Z-score ranging from 0.42 to 1.31. The Z-score of younger MDD patients notably deviates from the mean of the normative database. For instance, the Z-scores of the 20–29 and 30–39 age groups are more than one standard deviation. However, as the age of MDD participants increases, particularly those over 40 years old, their Z-score gradually decreases, approaching closer to the mean of the normative database (Table 3).

### 3.4. Pearson Correlations between HRV Parameters and Depression, Anxiety in the MDD Group

Negative correlations were observed between the Z-scores of lnLF and the total score of BDI-II (r = −0.217, *p* < 0.01), cognitive depression (*r* = −0.199, *p* < 0.01), and somatic depression (*r* = −0.165, *p* < 0.05). Similarly, negative correlations were found between the Z-scores of the lnLF/HF ratios and the total score of BDI-II (*r* = −0.215, *p* < 0.01), cognitive depression (*r* = −0.180, *p* < 0.01), and somatic depression (*r* = −0.213, *p* < 0.01). Additionally, negative correlations were noted between the Z-scores of lnTP and the total score of BDI-II (*r* = −0.141, *p* < 0.05) and cognitive depression (*r* = −0.140, *p* < 0.05). These results suggest that higher depression scores correspond to lower HRV in patients with MDD (Table 4).

Furthermore, negative correlations were observed between the Z-scores of lnLF and the total score of BAI (*r* = −0.121, *p* < 0.05), as well as somatic depression (*r* = −0.122, *p* < 0.05). Similarly, negative correlations were found between the Z-scores of the lnLF/HF ratios and the total score of BAI (*r* = −0.150, *p* < 0.05), as well as cognitive anxiety (*r* = −0.156, *p* < 0.05). These findings indicate that higher anxiety symptoms are associated with lower HRV in patients with MDD (Table 5).

## 4. Discussion

This study reveals a gradual decline in HRV indices with advancing age in the HC group. Additionally, females in the HC group exhibit higher RMSSD and HF values than males. Conversely, patients in the MDD group demonstrate lower HRV indices than those in the HC group, with their symptoms of depression and anxiety showing a negative correlation with HRV indices. 

Among healthy controls, HRV indices show a gradual decrease with age, consistent with findings from previous studies employing both short-term five-minute HRV measurements [10,11,12,13] and 24 h long-term HRV measurements [29]. Furthermore, females display higher parasympathetic nervous system indicators (RMSSD and HF of HRV) compared to males, consistent with prior studies indicating elevated HF in females relative to males [10,30]. This higher parasympathetic nervous system activity in females may signify a better cardio-protection mechanism [10]. Comparing the SDNN and RMSSD values of short-term HRV indices in this study (41.95 ms and 31.41 ms, respectively) to prior studies, we find similarities with Baek et al.’s [11] findings among Koreans (44.73 ms and 28.68 ms, respectively), but discrepancies with Nunan et al.’s [9] systematic review across various populations (50 ms and 42 ms, respectively), as well as Ortega et al.’s [13] study in Singapore. These variations may stem from ethnic differences (Caucasians vs. Asians) and geographic locations (American and Asian). 

The healthy participants in this study comprised adults in various stages of aging. In contrast, some studies contain normative databases spanning a wide age range, from children to aging, with participants aged between 8 and 69 years [11], 10 and 80 years [29], and 10 and 89 years [13]. The age range in our study was limited to adults over the age of 20. This aligns with previous studies that predominately enrolled adult participants [9,12], as children, still in the development stage, often exhibit faster heart rates that may influence HRV indices [31]. 

In our cross-validity sample of patients with MDD, Z-scores were between −0.92 and 1.28, and the absolute value Z-score was between 0.42 and 1.31. The findings revealed that some patients with MDD exhibited Z-scores exceeding one standard deviation (SD). Lower HRV indices, such as lnLF, the lnLF/HF ratio, and lnTP, were associated with increased depressive symptoms. Additionally, decreased lnLF of HRV is linked to heightened anxiety symptoms, particularly somatic anxiety. These results align with Borrione et al.’s [32] findings, which demonstrated a negative correlation between depressive symptoms and LF of HRV and a positive correlation with the LF/HF ratio. These data suggest vagal withdrawal in patients with MDD [14,17,19]. Additionally, the HRV indices demonstrated a decline with age in both the MDD and HC groups [28]. This study found large variation in younger groups (20–29, 30–39, 40–49) compared to older groups (50–59 and 60–69). Possible explanations include as follows: (1) Age-related decline in HRV indices and cardiac autonomic function, leading to convergence of HRV indices. (2) Among younger MDD participants, some exhibit depressive symptoms like low energy and fatigue, resulting in decreased HRV, while others experience anxiety, irritability, hyperarousal, and heightened activity levels, leading to HRV indices surpassing the mean ± 1.96 SD range. We have addressed this issue on page 5.

Several limitations were identified in this study. First, although we rigorously screened healthy adults without physical illnesses or mental disorders, the sample size in the HC group still needs to be increased. Second, the study aimed for a minimum of 30 participants per group, evenly distributed between genders. Although each age group included at least 30 healthy participants, some age groups had a shortage of males, notably the 30–39, 50–59, and 60–69 groups. Fewer healthy males without physical illnesses or mental disorders were willing to participate in the study, and it was challenging to recruit this group of healthy males around us. Consequently, compromising the representativeness of these age groups may be insufficient. Third, antidepressants may influence the HRV indices [33]; patients with MDD tend to combine different types of antidepressants. Therefore, this study only compared the deviation of the Z-score from the mean values of the HC group. Fourth, ECG measurements conducted between 9:00 and 17:00 may not capture the full spectrum of HRV variations; HRV indices were influenced by the effect of endogenous circadian rhythms and ultradian components on participants’ autonomic nervous system and HRV indices.

## 5. Conclusions

The HRV normative database crosses 20–70 year olds in Taiwan, and the HRV indices gradually decline with advancing age in the HC group; females exhibit higher parasympathetic activity than males. Patients with MDD demonstrated lower HRV indices than those in the HC group, with their symptoms of depression and anxiety showing a negative correlation with HRV indices. The Taiwan HRV normative database has good psychometric characteristics of cross-validation.

## Figures and Tables

**Table 1:** The HRV normative database.

Authors	Nunan [9] (Review Article)	Abhishekh [10]	Baek [11]	Choi [12]	Ortega [13]	Chang (This Study)
Country	From 44 studies	India	South Korea	South Korea	Singapore	Taiwan
Years of data collection/measure time	January 1997 to September 2008			9:00 to 18:00	November 2021 to December 2022	2013–2023 9:00 to 18:00
Sample size	21,438	189	467	291	2143	311
Sex (Male/Female)		114M/75F	249M/218F	144M/147F	974M/1,169F	137M/174F
Age (years)	>18	16–60	8–69	19–69	10–89	20–70
Age group	NA	16–20, 21–30, 31–40, 41–50, and 51–60	10, 20, 30, 40, 50, and 60	19–29, 30–39, 40–49, 50–59, and 60–69	10–11, 12–19, 20–29, 30–39, 40–49, 50–59, 60–69, 70–79, and 80–89	20–29, 30–39, 40–49, 50–59, and 60–69
Equipment	NA	Power Lab	Freeze-Framer	Ubpulse T1	Elite HRV	Infiniti 6.0
Sensor/location	NA	Lead II ECG (1024 Hz/s) at supine position	PPG sensor/right index finger at sitting-rest	Pulse Analyzer/left index finger	Core Sense of PPG sensor/right index finger	Lead II ECG (2048 Hz/s) at sitting-rest
Short time HRV	5 min	5 min	5 min	5 min	5 min	5 min
Controlling breathing rates	Breathe naturally/paced	12–15 breaths per minute	Breathe naturally	Breathe naturally	Breathe naturally	Breathe naturally
HRV indices	HR, SDNN, RMSSD, LF, HF, LF.nu, HF.nu, LF/HF	SDNN, RMSSD, LF, HF, LF.nu, HF.nu, LF/HF	HR, SDNN, RMSSD, pNN50, LF, HF, LF.nu, HF.nu, LF/HF	SDNN, pNN50 VLF, LF, HF, TP, HRV triangular index (HRV index)	HR, SDNN, RMSSD	SDNN, RMSSD, LF, HF, LF/HF, TP
SDNN	50 ± 16		44.73 ± 27.51	M: 31.31 ± 11.69/ F: 32.07 ± 12.27	52.0 ± 19.5	41.95 ± 16.26
RMSSD	42 ± 15		28.68 ± 18.95		42.4 ± 17.0	31.41 ± 16.06

Note: ECG, electrocardiographic; HF, high frequency; HF.nu, normalized high frequency; LF, low frequency; LF.nu, normalized low frequency; NA, not applicable; PPG, photoplethysmography; RMSSD, root mean square of the successive normal-to-normal interval differences; SDNN, standard deviation of normal-to-normal intervals; TP, total power; VLF, very low frequency.

**Table 2:** Demographic characteristics, psychological questionnaires, and the HRV differences between the HC and MDD groups.

	HC Group (*n* = 311)	MDD Group (*n* = 272)			
	M ± SD	M ± SD	*t*/*X*^2^/*F*	*p*	*np* ^2^
Age	39.29 ± 14.21	39.69 ± 14.03	−0.349	0.728	
Sex					
Male *n*, %	137, 44.1%	66, 24.3%	25.030 ***	<0.001	
Female *n*, %	174, 55.9%	206, 75.7%			
Total score of BDI-II ^a^	4.4 ± 3.56	31.95 ± 10.56	1772.693 ***	<0.001	0.753
Cognitive depression ^a^	2.55 ± 2.57	23.81 ± 8.95	1517.665 ***	<0.001	0.724
Somatic depression ^a^	1.85 ± 1.64	8.14 ± 3.11	907.746 ***	<0.001	0.610
Total score of BAI ^a^	1.95 ± 1.92	22.03 ± 9.92	1142.953 ***	<0.001	0.663
Cognitive anxiety ^a^	0.89 ± 1.29	13.53 ± 5.67	1370.366 ***	<0.001	0.703
Somatic anxiety ^a^	1.29 ± 1.69	8.84 ± 6.05	405.975 ***	<0.001	0.412
SDNN (ms) ^a^	41.95 ± 16.26	32.99 ± 15.91	40.207 ***	<0.001	0.065
RMSSD (ms) ^a^	31.41 ± 16.06	25.65 ± 16.59	21.298 ***	<0.001	0.035
lnLF (ms^2^/Hz) ^a^	4.68 ± 1.15	3.93 ± 1.31	44.932 ***	<0.001	0.072
lnHF (ms^2^/Hz) ^a^	4.63 ± 1.25	4.05 ± 1.36	31.836 ***	<0.001	0.052
lnLF/HF ratio ^a^	0.06 ± 1.06	−0.12 ± 1.07	0.630	0.428	0.001
lnTP (ms^2^/Hz) ^a^	6.14 ± 0.88	5.56 ± 1.04	47.656 ***	<0.001	0.076
Antidepressants ^b^ *n*, %		247, 93.56%			
SSRI *n*, %		103, 39.0%			
SNRI *n*, %		50, 18.93%			
TCA *n*, %		11, 4.16%			
other antidepressants *n*, %		118, 44.69%			
Benzodiazepines *n*, %		213, 80.68%			
non-benzodiazepines *n*, %		56, 21.21%			
Antipsychotic *n*, %		78, 29.54%			
Antiepileptic *n*, %		12, 4.54%			

*** *p* < 0.001. ^a^ ANCOVA was used to control for covariance (sex) and compared group differences in psychological questionnaires and HRV indices between the HC and MDD groups. ^b^ Eight patients in the MDD group were free of medications; statistical analysis of medications from 264 patients.

**Table 3:** The HRV difference between males and females in the HC and MDD groups.

	Female	Male			
The HC group	M ± SD (*n* = 174)	M ± SD (*n* = 137)	*F*	*p*	*np* ^2^
SDNN (ms)	42.30 ± 15.87	41.50 ± 16.80	0.187	0.666	0.001
RMSSD (ms)	33.96 ± 17.23	28.17 ± 13.83	10.260 **	0.002	0.032
lnLF (ms^2^/Hz)	4.63 ± 1.04	4.76 ± 1.26	0.980	0.323	0.003
lnHF (ms^2^/Hz)	4.80 ± 1.19	4.41 ± 1.29	7.657 **	0.006	0.024
lnLF/HF ratio	−0.17 ± 1.01	0.35 ± 1.05	19.589 ***	<0.001	0.060
lnTP (ms^2^/Hz)	6.15 ± 0.82	6.13 ± 0.95	0.064	0.801	0.000
The MDD group	M ± SD (*n* = 206)	M ± SD (*n* = 66)	*F*	*p*	*np* ^2^
SDNN (ms)	31.78 ± 14.60	36.79 ± 19.05	5.040 *	0.026	0.018
RMSSD (ms)	25.36 ± 16.48	26.54 ± 17.03	0.253	0.616	0.001
lnLF (ms^2^/Hz)	3.80 ± 1.27	4.35 ± 1.35	8.998	0.003	0.032
lnHF (ms^2^/Hz)	4.06 ± 1.32	4.04 ± 1.50	0.016	0.899	0.000
lnLF/HF ratio	−0.26 ± 1.02	0.31 ± 1.14	14.865 ***	<0.001	0.052
lnTP (ms^2^/Hz)	5.50 ± 0.99	5.76 ± 1.16	3.154	0.077	0.012

Note: * *p* < 0.05. ** *p* < 0.01. *** *p* < 0.001.

**Table 4:** Z-scores and absolute value Z-scores of HRV in the MDD group.

Age Group (Years)	Total (*n*)	Male/Female (*n*)	SDNN M ± SD	RMSSD M ± SD	lnLF M ± SD	lnHF M ± SD	lnLF/HF Ratio M ± SD	lnTP M ± SD
Z-scores								
20–29	89	25/64	−0.64 ± 1.16	−0.43 ± 1.09	−0.78 ± 1.41	−0.53 ± 1.30	−0.15 ± 1.03	−0.82 ± 1.46
30–39	45	8/37	−0.70 ± 1.14	−0.50 ± 1.21	−0.91 ± 1.37	−0.90 ± 1.50	−0.08 ± 1.14	−0.92 ± 1.45
40–49	64	15/49	−0.60 ± 0.97	−0.19 ± 0.96	−0.56 ± 1.01	−0.30 ± 0.91	−0.16 ± 1.12	−0.84 ± 1.19
50–59	47	11/36	−0.37 ± 0.72	−0.24 ± 0.76	−0.51 ± 0.95	−0.15 ± 0.91	−0.17 ± 0.77	−0.30 ± 0.91
60–69	27	7/20	1.11 ± 0.71	1.00 ± 0.61	1.27 ± 0.98	1.02 ± 0.96	−0.46 ± 1.25	1.28 ± 1.08
Total	272	66/206						
Absolute value Z-scores								
20–29	89	25/64	1.11 ± 0.71	1.00 ± 0.61	1.27 ± 0.98	1.02 ± 0.96	0.84 ± 0.61	1.28 ± 1.08
30–39	45	8/37	1.16 ± 0.64	1.01 ± 0.82	1.29 ± 1.02	1.28 ± 1.19	0.89 ± 0.71	1.31 ± 1.11
40–49	64	15/49	0.91 ± 0.53	1.01 ± 0.83	1.07 ± 0.82	1.02 ± 0.78	0.9 ± 0.67	1.12 ± 0.84
50–59	47	11/36	0.96 ± 0.60	0.67 ± 0.71	0.93 ± 0.68	0.77 ± 0.56	0.63 ± 0.46	1.16 ± 0.86
60–69	27	7/20	0.71 ± 0.37	0.66 ± 0.43	0.86 ± 0.65	0.75 ± 0.51	1.14 ± 0.67	0.76 ± 0.58
Total	272	66/206						

**Table 5:** The correlations between Z-scores of HRV indices, depression, and anxiety in the MDD group.

MDD	Z_SDNN	Z_RMSSD	Z_lnLF	Z_lnHF	Z_lnLF/HF Ratio	Z_lnTP
Total score of BDI-II	−0.113	0.009	−0.217 **	−0.042	−0.215 **	−0.141 *
Cognitive depression	−0.112	0.011	−0.199 **	−0.040	−0.180 **	−0.140 *
Somatic depression	−0.061	0.000	−0.165 **	−0.025	−0.213 **	−0.074
Total score of BAI	−0.025	0.055	−0.121 *	−0.015	−0.150 *	−0.048
Cognitive anxiety	−0.032	0.048	−0.089	0.025	−0.156 *	−0.031
Somatic anxiety	−0.015	0.048	−0.122 *	−0.043	−0.111	−0.063

Note: * *p* < 0.05. ** *p* < 0.01.

## Data Availability

Original data are available upon request.
